# A Novel Cold-Adaptive Endo-1,4-β-Glucanase From *Burkholderia pyrrocinia* JK-SH007: Gene Expression and Characterization of the Enzyme and Mode of Action

**DOI:** 10.3389/fmicb.2019.03137

**Published:** 2020-01-22

**Authors:** Feifei Chen, Jianren Ye, Ayyappa Kumar Sista Kameshwar, Xuelian Wu, Jiahong Ren, Wensheng Qin, De-Wei Li

**Affiliations:** ^1^Jiangsu Key Laboratory for Prevention and Management of Invasive Species, Co-innovation Center for Sustainable Forestry in Southern China, College of Forestry, Nanjing Forestry University, Nanjing, China; ^2^Department of Biology, Lakehead University, Thunder Bay, ON, Canada; ^3^Department of Biology Science and Technology, Changzhi College, Changzhi, China; ^4^The Connecticut Agricultural Experiment Station, Valley Laboratory, Windsor, CT, United States

**Keywords:** cloning, expression, GC-rich gene, endo-1, 4-β-glucanase, *Burkholderia pyrrocinia*, cold-adaptive enzyme

## Abstract

The efficient industrial conversion of plant-derived cellulose to simple sugars and other value-added chemicals requires various highly stable and reactive enzymes. Industrial processes especially synchronous saccharification and fermentation (SSF)-based production of cellulosic bio-ethanol require enzymes that are active at lower temperatures. In this study, we have identified, characterized, and expressed the cold-adaptive endo-1,4-β-glucanase (BpEG) isolated from the *Burkholderia pyrrocinia* JK-SH007. The analysis of the predicted amino acid sequence indicated that BpEG belongs to GH family 8. The *BpEG* without the signal peptide was cloned into the expression vector pET32a and significantly expressed in *Escherichia coli* BL21 (DE3) competent cells. The SDS-PAGE and Western blot analysis of BpEG revealed that the recombinant BpEG was approximately 60 kDa. Purified recombinant BpEG exhibited hydrolytic activity against carboxymethyl cellulose (CMC) and phosphoric acid swollen cellulose (PASC), but not crystalline cellulose and xylan substrates. High performance, anion exchange, chromatography-pulsed amperometric detector (HPAEC-PAD) analysis of the enzymatic products obtained from depolymerization of 1,4-β-linked biopolymers of different lengths revealed an interesting cutting mechanism employed by endoglucanases. The recombinant BpEG exhibited 6.0 of optimum pH and 35°C of optimum temperature, when cultured with CMC substrate. The BpEG enzyme exhibited stable activity between pH 5.0 and 9.0 at 35°C. Interestingly, BpEG retained about 42% of its enzymatic activity at 10°C compared to its optimal temperature. This new cold-adaptive cellulase could potentially achieve synchronous saccharification and fermentation (SSF) making BpEG a promising candidate in the fields of biofuel, biorefining, food and pharmaceutical industries.

## Graphical Abstract

BpEG randomly cleaves cellulose polymers in low temperature.

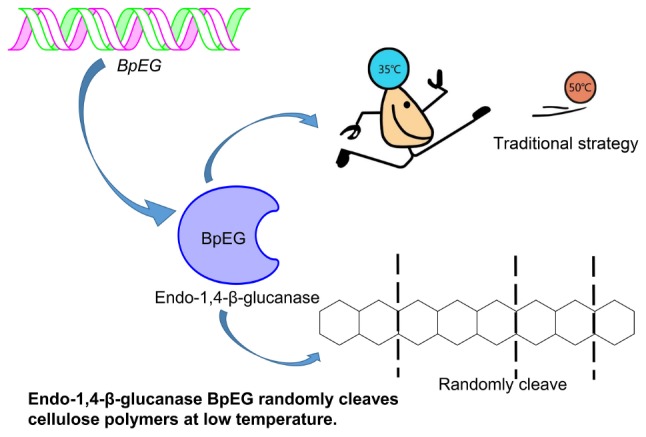

## Introduction

Increasing global population and continuous depletion of fossil fuels worldwide have forced the search for renewable fuel alternatives (Zhao et al., 2011; Rafique and Rehman, 2017). Incipient cellulosic-ethanol production methods based on food crops like corn played a certain but short-term role. However, the food versus fuel debate and continuously increasing demand for food supplies around the world lead to second-generation fuels. Although the second-generation fuels overcame the disadvantages of first-generation fuels by using cheaper, non-food crops as the substrate for fuel production, the usage of fertile land to produce energy crops is a significant obstacle. These disadvantages have led to the development of promising third- and fourth-generation biofuels that use wood, agricultural, algal residues, and other new technologies based on the simultaneous conversion of carbon dioxide (Balat, 2011).

Cellulose is one of the major components of plant biomass (lignocellulose), one of the most abundant organic components in nature, and a remarkable renewable energy resource (Heinze et al., 2018). Efficient breakdown and conversion of complex cellulose into simple glucose is a crucial process in biofuel generation and yet a major hurdle in the production of cellulosic ethanol. Many bacterial and fungal strains secrete highly catalytic cellulases, which efficiently break down the 1,4-β-glycosidic linkages of cellulose resulting in linear polymers of hydro glucopyranoses (Bianchetti et al., 2013; Wang et al., 2014). In nature, enzymatic hydrolysis of cellulose to glucose is a complicated process, because of its rigid and harsh structure which requires a synergistic action of cellulolytic enzymes including exo-β-1,4-glucanases (EC3.2.1.91), endo-β-1,4-glucanases (EC3.2.1.4), and β-glucosidases (EC 3.2.1.21) (Sharma et al., 2016). Firstly, glucose and cellobiose are released from the terminals of cellulose polymers by exo-β-1,4-glucanases (EC3.2.1.91). Secondly, the internal structures of the biopolymers connected through 1,4-β-glycosidic bonds are converted to cello-oligosaccharides of assorted lengths by endo-β-1,4-glucanases (EC3.2.1.4) by exhibiting a random degradation pattern (Park and Yun, 1999). Finally, the 1,4-β-glucosidase (EC3.2.1.21) produces glucose from its cleavage of cello-oligosaccharides. Among these enzymes, endo-β-1,4-glucanase (EC 3.2.1.4) is extremely important as it cleaves the β-1,4-glycosidic bonds of cellulose and generates cello-oligosaccharides of random length (Huang et al., 2016). Thus, several studies have been conducted to develop and characterize recombinant endo-β-1,4-glucanases from various microbial sources. In particular, the endo-β-1,4-glucanases active at lower temperatures could potentially be of higher importance industrially, offering low energy processes that are both cost and time-efficient.

The industrial process of converting lignocellulosic plant biomass to a biofuel substrate primarily involves pre-treatment for removing lignin (Uppugundla et al., 2014), enzymatic hydrolysis of cellulose into sugar (Silveira et al., 2014), and ethanol fermentation using yeasts (Wu et al., 2016). Conversion of cellulose to bioethanol is a two-step process: saccharification and fermentation, respectively. The saccharification process is conducted at higher temperatures between 50 and 55°C (which requires high energy and cost) (Murashima et al., 2002; Wang et al., 2009; Liu et al., 2016), while the ethanol fermentation using *Saccharomyces cerevisiae* occurs at lower temperatures between 25 and 30°C (Borrull et al., 2016; Reis et al., 2018). Thus a cold-adaptive, endo-β-1,4-glucanase catalyzing the saccharification process under ambient temperatures could be of high significance. According to the report of Khalili Ghadikolaei et al. (2018), the endo-β-1,4-glucanases active under lower temperatures can enhance various industrial applications including paper-pulp processing, biofuel, food and feedstock, textile, laundry, and pharmaceutical industries (Khalili Ghadikolaei et al., 2018). Exploration of cold-adaptive cellulases provides a great potential to achieve synchronous saccharification and fermentation (SSF).

Research and applications of cold-active enzymes from *Burkholderia* have paved the way for our current study. A cold-active lipase isolated from *Burkholderia* sp. (SYBC LIP-Y) was characterized and overexpressed, which exhibited higher catalytic activities at lower temperatures (Yuan et al., 2010). Similar studies conducted by Jin et al. has reported an efficient cold-adapted lipase (LIP-BA) isolated from the *Burkholderia anthina* NT15, which exhibited higher catalytic activities even at lower temperatures (Jin et al., 2012). However, the study of endo-1,4-β-glucanase from *Burkholderia* is limited. To explore cold-adaptive cellulases could potentially achieve synchronous saccharification and fermentation (SSF). Thus, we report the heterologous expression of a novel cold-adaptive endo-1,4-β-glucanase gene isolated from *Burkholderia pyrrocinia*.

## Materials and Methods

### Bioinformatic Analysis

We have performed an automated NCBI Blastn and Blastx protein homology analysis using the sequence fragment which is annotated as an endo-1,4-β-glucanase from the whole genome sequence of the *B. pyrrocinia* JK-SH007 strain (as sequenced by Beijing Genomics Institute-BGI), and we further named the sequence fragment as *BpEG*. The BpEG amino acid sequence retrieved from the *B. pyrrocinia* JK-SH007 strain was aligned with the GH8 family proteins downloaded from the NCBI database using the ClustalW program. The signal peptide analysis of BpEG was performed using the SignalP 4.0 server website^1^. The above-obtained BpEG amino acid sequences were subjected to 3D homology modeling analysis using SWISS-MODEL online web-database and PC version software (SPDBV_4.10_PC). The homology modeling of BpEG was achieved using PDB (protein data bank) template protein with accession ID 3qxq.1.

### Cloning of the *BpEG* Gene

The full-length *BpEG* gene sequence was retrieved from the genomic DNA of *B. pyrrocinia* JK-SH007 using the designed forward (5′-ATGGCGAAGCGACGGGTAACG-3′) and reverse (5′-TCAGCGGGCGGCGCACGAAGG-3′) primers. The *BpEG* gene sequence we obtained (described above) was amplified using a PCR machine with the PCR reaction mixture containing *B. pyrrocinia* DNA (50-100 ng), 0.1 μM the specific primer, 80 μM dNTP, 0.5 U LA Taq and 10 μL 2 × GC buffer II (Takara Bio, Dalian, China). The PCR reaction was performed using the following conditions: 95°C for 10 min, thermocycling consisted of 33 cycles at 95°C for 10 s, 60°C for 10 s, and 72°C for 90 s, with a final extension at 72°C for 10 min. The PCR products were analyzed using 1% agarose gel. The resulting 1.2-kb DNA fragment was purified and cloned into a pMD^TM^19-T vector (Takara Bio, Dalian, China) thus, the obtained *BpEG* gene was subjected to DNA sequencing.

### Construction of the Expression Plasmid

The *BpEG* gene fragment without the signal peptide coding sequence was amplified using the custom-designed forward (5′-TGAATTCCGCGCGCAGGCCGCGGGCGCC-3′, *Eco*RI site underlined) and reverse (5′-CAAGCTTTCAGCGGG CGGCGCACGAAGG-3′, *Hin*dIII site) PCR primers. The PCR primers were synthesized in the regions corresponding to amino acid residues 33–39 and 399–405 of BpEG, respectively. The PCR reaction mixture (total of 20 μL Prime STAR buffer) contained *B. pyrrocinia* JK-SH007 DNA, 0.1 μM of each primer, 80 μM of each dNTP, 0.5 U of Takara PrimeSTAR DNA polymerase and PrimeSTAR GC buffer (Takara Bio, Dalian, China). The PCR cycle was set up for 33 cycles at 95°C for 30 s, 60°C for 30 s, and 72°C for 90 s, thus obtained PCR products were analyzed using 1% agarose gel. The resulting 1.1-kb DNA fragment was cloned into the pMD^TM^19-T vector by following the manufacturer’s instructions. The cloning vector (*BpEG*-pMD^TM^19-T) containing the DNA fragment and expression vector (pET32a) were treated with the restriction enzymes *Eco*RI and *Hin*dIII. The DNA fragments of *BpEG* and linearized pET32a vector carrying a thioredoxin-6 × His-tag were mixed and ligated with T4 DNA ligase (Takara Bio, Dalian, China). The expression plasmid coding a mature *BpEG* was named pET32a-*BpEG* and was transformed into *Escherichia coli* JM109 competent cells. The transformed colonies were evaluated using colony PCR followed by sequencing the *BpEG* gene.

### Heterologous Expression of Recombinant BpEG

The recombinant pet32a-*BpEG* expression plasmid carrying a thioredoxin-6 × His-tag was transformed into *E. coli* BL21 (DE3) competent cells. The transformed bacterium was cultured in 100 mL LB medium with 100 μg/mL ampicillin at 37°C. The transformed bacterium was cultured until it reached an optical density of 0.6 at 600 nm, respectively. Expression of the recombinant BpEG containing the thioredoxin-6 × His-tag was induced with 0.5 mM IPTG by incubating at 16°C overnight. Later, the bacterial cells were harvested using centrifugation at 10,000 × *g* for 20 min at 4°C. The above-obtained cells were resuspended in 10 mL of 20 mM Tris–HCl buffer (pH 7.0) containing a serine protease inhibitor (PMSF), sonicated, and centrifuged at 10,000 × *g* for 20 min at 4°C. The supernatant was applied to a His GraviTrap with Ni-IDA and equilibrated using 20 mM phosphate buffer (pH 8.0) containing 20 mM imidazole and 300 mM NaCl. BpEG was eluted over 20–300 mM imidazole gradient at gravity flow. The active fractions were desalted and concentrated on an Amicon^®^ Ultra-15 centrifugal filter device that was equilibrated with 20 mM sodium acetate buffer containing 100 mM NaCl at pH 5.0. The enzyme was eluted using 20 mM Tris–HCl (pH 7.0) to obtain a purified enzyme solution.

### SDS-PAGE and Western Blot Analysis

The molecular weight of the purified recombinant BpEG was determined using 12.5% sodium dodecyl sulfate-polyacrylamide gel electrophoresis (SDS-PAGE) (Laemmli, 1970) using the Opti-Protein Marker/Ladder (Applied Biological Materials, Inc., Canada). Protein bands were detected using Coomassie Brillant Blue (CBB) R-250 staining. The expression of BpEG protein samples was characterized using Western blotting with the standard protocols (Laemmli, 1970; Towbin et al., 1979). The purified BpEG protein was also loaded on to 12.5% sodium dodecyl sulfate-polyacrylamide gel electrophoresis (SDS-PAGE) and electroblotted for 1 h onto the polyvinylidene fluoride (PVDF) membrane in a Tris-glycine-methanol buffer. After, the membrane was incubated in a 1% bovine serum albumin (BSA) solution overnight and later probed with 6 × His-tag antibodies at 1:1,000 dilutions and a rabbit Anti-Mouse IgG (H&L) AP-conjugated with alkaline phosphatase was used as the secondary antibody at a dilution of 1:7,500 (Promega, United States). Chromogenic detection of BpEG protein bands was performed using western blue, a stabilized substrate for alkaline phosphatase (Promega, United States), and the reaction was later stopped by rinsing with water.

### Partial Peptide Fragment Sequence

To further identify the purified enzyme, the single band was cut out from the gel and digested with trypsin. The protein products were further analyzed and characterized using a liquid chromatography-electrospray, ionization-tandem mass spectrometry (LC-ESI-MS/MS). We have used an automated protein sequencer to determine the partial amino acid sequence of the BpEG peptide (Ueda et al., 2008).

### Enzyme and Protein Assay

The activity of BpEG was determined using the standard 3,5-dinitrosalicylic acid (DNS) method by determining the reduced sugars released from CMC and PASC substrates. The above reactions were continuously monitored at an absorbance of 540 nm (Miller, 1959). The one unit of endo-1,4-β-glucanase activity is determined as the enzyme required to produce the amount of reducing sugar equal to 1 μmol of glucose per min of reaction time. The protein concentration of BpEG was quantified using the BCA Protein Assay Kit. The whole experiment was conducted in triplicate (both the reaction and its control samples).

### Effects of pH and Temperature on BpEG Activity and Stability

The enzymatic activity of recombinant BpEG was determined using CMC (substrate) at a wide range of pH and temperatures. The optimal temperature required for recombinant BpEG was determined by culturing at a wide range of temperatures between 10 and 60°C with 5°C intervals, respectively. The effect of pH on enzyme activity was measured by culturing at different pH ranging between pH 3.0 and 11 with the temperature set at 35°C for 30 min. The pH stability of BpEG was determined by incubating the BpEG at 35°C for 30 min using 0.1 M of the following buffer solutions: sodium citrate (pH 3.0–6.0), sodium phosphate (pH 6.0–8.0), and glycine-NaOH (pH 9.0–11.0). Similarly, the temperature stability of the BpEG was determined by incubating in 0.1 M sodium citrate (pH 6.0) for 15–45 min at various temperatures ranging from 1 to 60°C. These experiments were conducted using the protocol reported by Ueda et al. (2014). The amount of reducing sugars produced by these reactions was further measured using the standard 3,5-dinitrosalicylic acid (DNS) method as described above (Miller, 1959). All the experiments were conducted in triplicates.

### Substrate Specificity

The substrate specificity of BpEG was determined by using the β-glycosidic bonds containing polymers such as microcrystalline cellulose (Avicel), PASC (Schulein, 1997), xylan, and CMC. The enzyme activity of BpEG was measured using the standard 3,5-dinitrosalicylic acid (DNS) method as described above (Miller, 1959) by calculating the amount of reducing sugars produced.

### HPAEC-PAD Analysis

The mode of action of the BpEG enzyme was determined using high-performance anion exchange chromatography equipped with a pulsed amperometric detector (HPAEC-PAD). The reaction mixture contained 0.1 M acetate buffer (pH 6.0) with 10 μL enzyme and 1 mgmL^–1^ of 1,4-β-linked CMC and oligosaccharides (CMC, cellopentaose, cellotetraose, or cellotriose). The reaction was carried out at 35°C, and the samples were collected after 24 h and further characterized by HPAEC-PAD as previously described by Xu et al. (2013).

### The Potential Application of BpEG

The potential application of BpEG was explored by degrading different biomass, which included wheat bran, oat grain, and ginkgo leaves. All the biomass was dried in an oven at 65°C till constant weight obtained and grounded in the mortar. Then the biomass was separated through the 0.149 millimeters sieve (100 series). In order to remove the background effects, the biomass was washed 5 times by using hot water at 65°C. The amount of reducing sugars produced by these reactions was measured using the standard 3,5-dinitrosalicylic acid (DNS) method, as described above (Miller, 1959). Determination of reducing sugars was performed under optimal conditions. All the experiments were conducted in triplicates.

### Statistical Analysis

The data was processed and analyzed using Origin 8.0 software while the image processing and layout was done using the Adobe illustrator CC 2017 software.

### Nucleotide Sequence Accession Number

The complete sequence of the endo-1,4-β-glucanase gene (*BpEG*) from *B. pyrrocinia* JK-SH007 is reported in the present paper and has been deposited in the NCBI database under the NCBI accession number MH733823.

## Results and Discussion

We have reported the isolation and characterization of the *B. pyrrocinia* JK-SH007 strain from the stems of *Populus deltoids.* This bacterium was characterized using the 16S rDNA sequence analysis, recA gene sequence analysis combined with morphological, physiological, biochemical characteristics, and the Biolog^®^ identification system, which revealed the isolated *B. pyrrocinia* JK-SH007 strain as part of the Bcc (Ren et al., 2011). In the present study, we identified, cloned and expressed the extrinsic endo-1,4-β-glucanase gene isolated from the *B. pyrrocinia* JK-SH007 strain.

### Cloning and Sequence Analysis of *BpEG*

The protein sequence homology analysis based on NCBI Blastx and Blastn resulted in an ideal candidate exhibiting the highest similarity for endo-1,4-β-glucanase and we named the DNA sequence fragment as *BpEG*. Further sequence-based SignalP analysis revealed that BpEG has a signal peptide with 32 amino acid residues at the N-terminus. Moreover, the gene sequence analysis showed that the *BpEG* gene contains an open reading frame (ORF) of 1,218 nucleotides capable of encoding a polypeptide of 405 amino acids. The high percentage of G and C (74.71% GC) nucleotides in *BpEG* sequence was the major challenge in conducting the gene cloning experiment. This could be one of the reasons for the absence of *Burkholderia* endo-1,4-β-glucanase gene cloning reports. The complete *BpEG* gene sequence was deposited in the GenBank database with the accession number MH733823.

**FIGURE 1:**
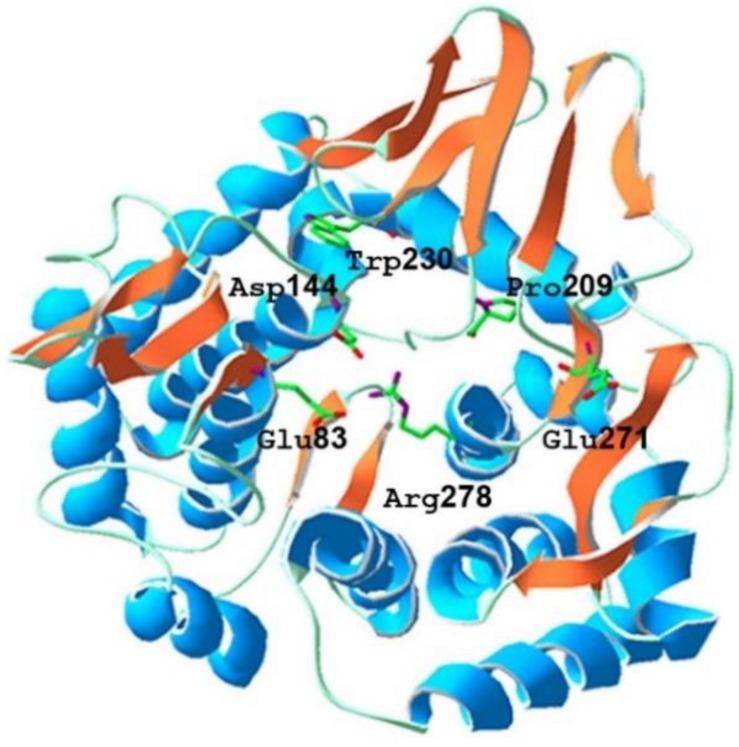
The predicted tertiary structure of *Burkholderia pyrrocinia* JK-SH007 BpEG. The catalytic glutamic acid residues and other conserved residues of GH8 are indicated in the above structure.

**FIGURE 2:**
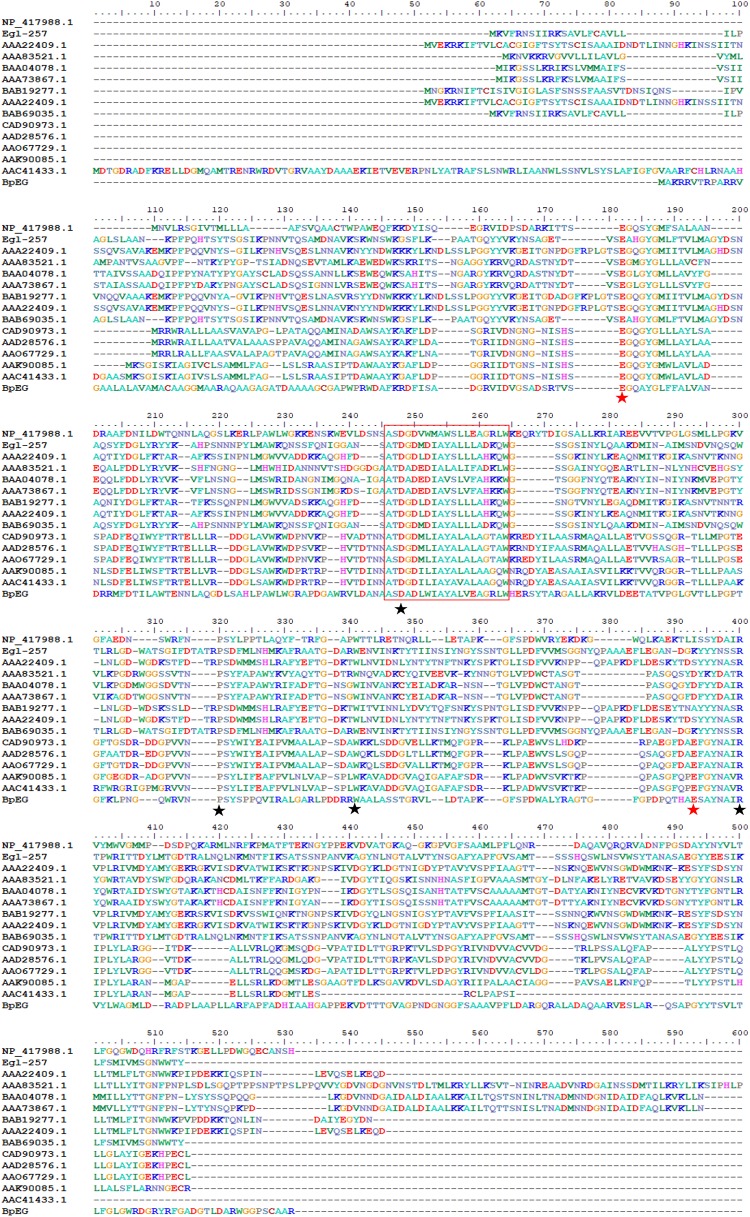
Amino acid sequence alignment of BpEG from *B. pyrrocinia* JK-SH007 with the family GH8 from *Escherichia coli* K-12 (NP_417988.1), *Bacillus circulans* KSM-N257 (Egl-257) (Hakamada et al., 2002), *Bacillus* sp. KK-1 (AAC27700.1), *Clostridium thermocellum* NCIB 10682 (AAA83521.1), *Clostridium josui* FERM P-9684 (BAA04078.1), *Clostridium cellulolyticum* ATCC 35319 (AAA73867.1), *Bacillus* sp. No.7-M (BAB19277.1), *Bacillus* sp. KSM-330 (AAA22409.1), *Bacillus circulans* KSM-N257 (BAB69035.1), *Rhizobium leguminosarum* bv. *trifolii* ANU843 (CAD90973.1), *Rhizobium leguminosarum* bv. *trifolii* R201 (AAD28576.1), *Rhizobium leguminosarum* bv. *trifolii* 1536 (AAO67729.1), *Agrobacterium tumefaciens* C58 (Cereon) (AAK90085.1), and *Agrobacterium tumefaciens* C58 (U. North Carolina) (AAC41433.1) using the ClustalW program. The highly conserved residues and catalytic residues are indicated with black and red asterisks, respectively. Conservative motif was in red box.

The sequence similarity analysis showed that BpEG displayed similarity with the endo-1,4-β-glucanase of *Burkholderia stabilis* (97%; WP_096470653), *Burkholderia cenocepacia* (92%; WP_053524826), and *Burkholderia contaminans* (92%; WP_046545415). These *Burkholderia* 1,4-β-glucanases all belonging to the glycoside hydrolase (GH) family 8 suggest that BpEG might also belong to the GH 8 family. Six strictly conserved residues of GH8 Glu 83, Asp144, Pro209, Trp230, Glu271, and Arg278 (Figure 1) were found in the catalytic module of BpEG according to the multiple sequence alignment (Figure 2) as described by Adachi et al. (2004) and Na et al. (2015). The amino acids sequencing analysis of BpEG using the ClustalW program revealed the conservative motif ASDADLWIAYALVEAGRLW (Figure 2).

The tentative 3-D homology model of BpEG developed using the SwissDock automated server showed that the BpEG structure encompasses a catalytic domain and a signal peptide like that of the endo-1,4-β-glucanase sequences of the *E. coli* (Accession Number: NP_417988.1) and *Bacillus circulans* (Park and Yun, 1999; Hakamada et al., 2002) as well as metagenomics study from Ladakh soil, as described previously by Bhat et al. (2013). The BpEG model structure also showed the putative catalytic residues, Glu83, and Glu271, correspond to the proton donor and acceptor (Figure 1), similar to the structure of *Bacillus* sp. K17 (Adachi et al., 2004). BpEG structure also showed high activity against the soluble substrate whereas it exhibited no catalytic activity against a crystalline cellulose substrate. It has also been suggested that the BpEG sequence does not contain a cellulose-binding domain.

### Heterologous Expression of Recombinant BpEG

**FIGURE 3:**
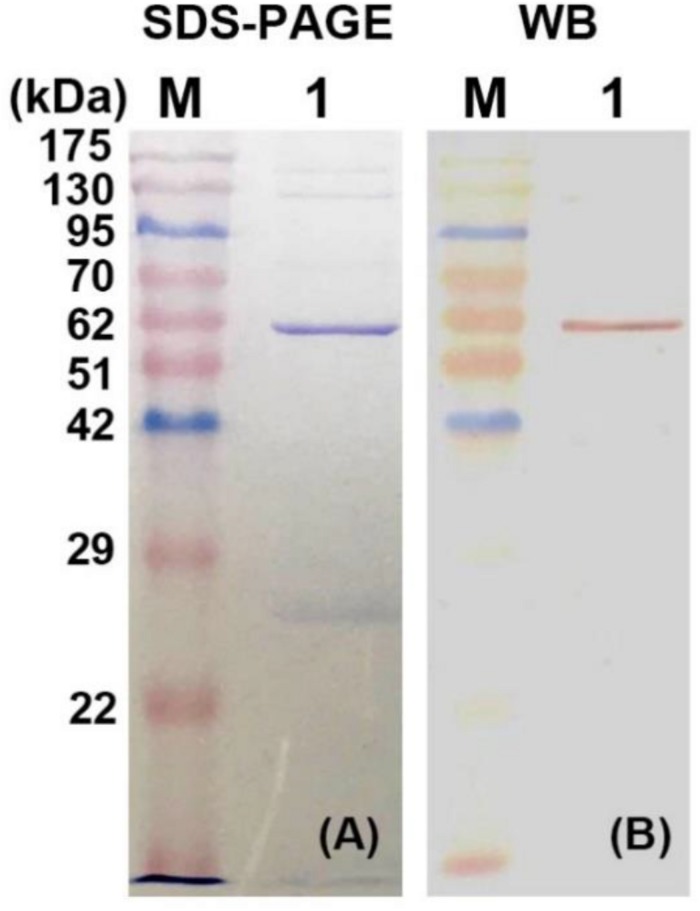
Expression and purification of recombinant BpEG. **(A)** The SDS-PAGE analysis of purified recombinant BpEG. **(B)** Western blot analysis of purified recombinant BpEG. M, opti-protein marker/ladder (Applied Biological Materials, Inc., Canada); 1, purified endo-1,4-β-glucanase.

The recombinant plasmid pET32a-*BpEG* was transformed into the competent cells of *E. coli* BL21 (DE3) and this expression of recombinant BpEG was induced by the IPTG. The activity of the endo-1,4-β-glucanase of the recombinant crude enzyme solution was determined to be 7.09 U/ml. The recombinant BpEG was purified from the culture supernatant of *E. coli* by Ni-IDA affinity chromatography. Further, we conducted the SDS-PAGE analysis under denaturing conditions, and the molecular weight of the purified recombinant BpEG was estimated to be 60 kDa (Figure 3A). The molecular weights of the endo-1,4-β-glucanases from other species, namely *Rhizopus oryzae* (Murashima et al., 2002), *Citrobacter farmeri* A1 (Bai et al., 2016), and *Ganoderma lucidum* (Liu et al., 2016), were previously reported.

### Western Blot Analysis and Identification of the Partial Peptide Fragment

Further characterization of the purified recombinant BpEG protein was performed using the Western blot analysis. The purified enzyme was initially separated using 12.5% SDS-PAGE and blotted onto a polyvinylidene fluoride (PVDF) membrane in Tris-glycine-methanol buffer. Results obtained from the Western blot analysis showed a band at 60 kDa. This result was found to be consistent with the SDS-PAGE results (Figure 3B). The sequencing analysis of the above-obtained recombinant BpEG fragments using an automatic protein sequencer showed us the conserved domains AIRVYLWAGM and VPGLGVTLLP. These conservative fragments exhibited high similarity with the endo-1,4-β-glucanases sequences of *E. coli* (Park and Yun, 1999) and *Citrobacter farmeri* A1 (Bai et al., 2016), which belong to GH 8.

### Properties of Recombinant BpEG

In order to study the catalytic properties of recombinant BpEG, we conducted a series of enzymatic assays with varying conditions, especially pH and temperatures, to study the stability of the recombinant BpEG protein. The recombinant BpEG exhibited the highest enzymatic activity toward CMC (100%), followed by PASC (62.5%), respectively. But BpEG cannot degrade crystalline cellulose and xylan (Table 1). These results suggested that BpEG was specific and had endoglucanases activity.

**TABLE 1:** The substrate specificity of recombinant BpEG.

**Item**	**Substrate**	**Relative activity**	**Production**
Polysaccharide polymer	CMC	100%	Glucose
	PASC	56%	Glucose
	Avicel	0	NO
	Xylan	0	NO
Oligosaccharide	Cellopentaose	ND	Cellotriose, cellotriose, cellotetraose, glucose
	Cellotetraose	ND	Cellotriose, glucose, cellobiose
	Cellotriose	ND	Cellotriose
	Cellobiose	ND	Cellobiose

*ND, not determined; NO, not obtained.*

**FIGURE 4:**
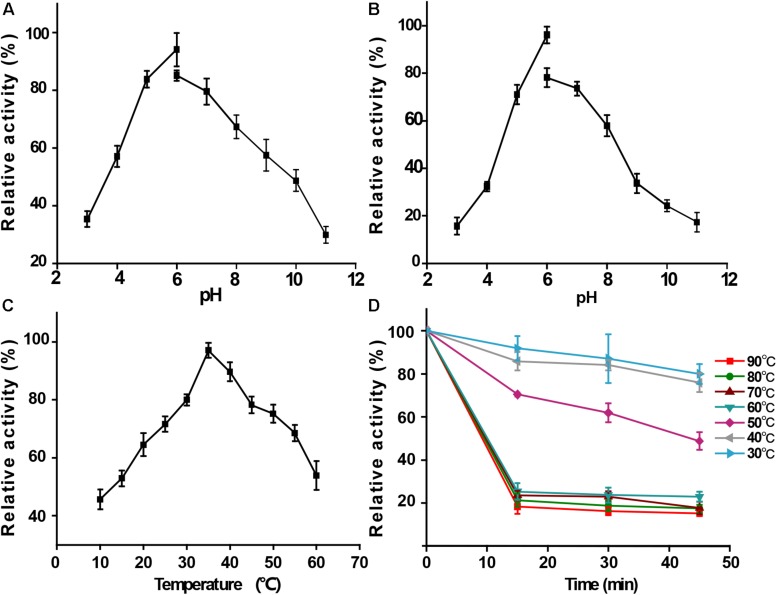
Functional properties of purified recombinant BpEG. **(A)** Effect of pH on enzyme activity at 35°C. **(B)** Effect of pH on enzyme stability at 35°C. **(C)** Effect of temperature on enzyme activity measured at 10–60°C. **(D)** Effect of temperature on enzyme stability after 15-45 min incubation at 40–90°C. The bars represent standard deviations.

**FIGURE 5:**
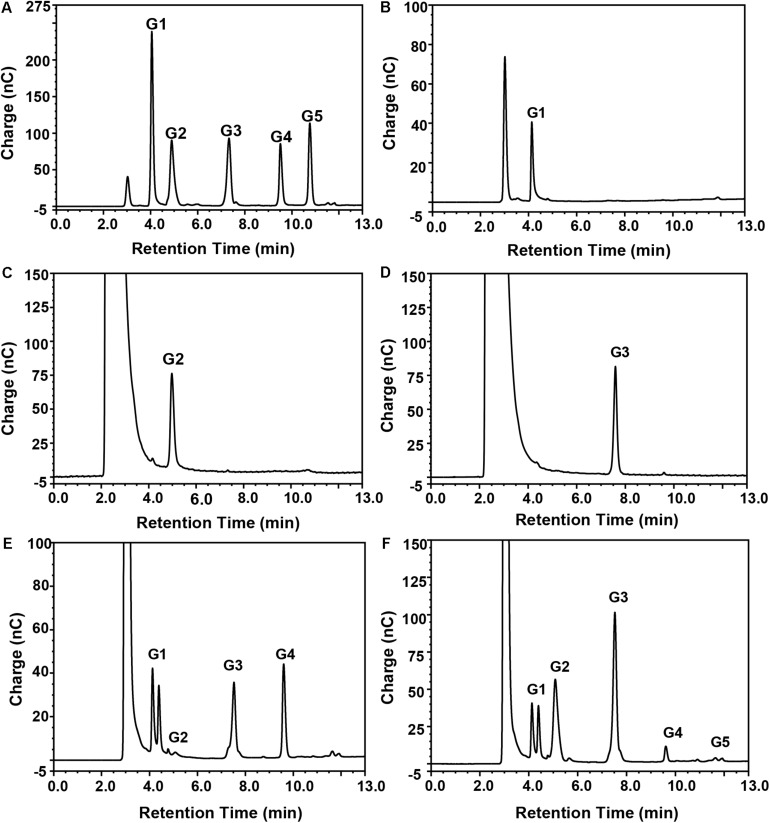
Mode of action of purified recombinant BpEG analyzed by HPAEC-PAD. Separation of the reaction products from enzyme reactions conducted with **(B)** CMC-Na, **(C)** cellobiose, **(D)** cellotriose, **(E)** cellotetraose, or **(F)** cellopentaose, as described in section “Materials and Methods.” Standards **(A)** G1, glucose; G2, cellobiose; G3, cellotriose; G4, cellotetraose; and G5, cellopentaose.

The CMC (Figure 5B) was used as the substrate in the subjected enzymatic assays to determine the functional properties of the recombinant BpEG protein. Results obtained from these enzymatic assays exhibited an optimum pH of 6 for the recombinant BpEG (Figure 4A and Table 2). The recombinant BpEG exhibited 70% of the maximum activity between a pH range of 5.0 and 7.0 (Figure 4B). The optimum pH of the recombinant BpEG protein showed comparable results with the previously reported endo-β-1,4 glucanases from *Rhizopus oryzae* (Murashima et al., 2002), *Eisenia foetida* (Ueda et al., 2014), and *G. lucidum* (Liu et al., 2016), respectively. However, another study reported that endo-1,4-β-glucanases isolated from the *Citrobacter farmeri* A1 showed an optimum pH of 3.5–7.5 (Bai et al., 2016).

**TABLE 2:** Properties of endo-1,4-β-glucanases.

**Origin (enzyme name)**	**Molecular mass (kDa)**	**Optimum pH**	**Optimum temperature (°C)**	**pH stability**	**Thermostability**	**References**
*Burkholderia pyrrocinia* JK-SH007 (BpEG)^∗^	43	6.0	35	5.0–8.0	30–50°C	This study
*Rhizopus oryzae* (RCE1)^∗^	41	6.0	55	4.0–6.0	55–65°C	Murashima et al., 2002
*Penicillium decumbens* (rPdCel5C)^∗^	67.6	4.8	40–50	4.0–5.0	30–50°C	Liu et al., 2013
*Citrobacter farmeri* A1 (EglC22b)^∗^	42	7.0	30–40	3.5–7.5	20–50°C	Bai et al., 2016
*Eisenia foetida* (EF-EG2)*^∗^*	50	5.5	40	5.0–9.0	∼40°C	Ueda et al., 2014
*Bacillus subtilis* JA18 (Egl499)	50	6.0	60	ND	ND	Wang et al., 2009
*Ganoderma lucidum* (GlCel5A)	43	3.0–4.0	60	3.0–4.0	ND	Liu et al., 2016
*Penicillium purpurogenum* (KJS506)	37	5.0	70	5.0-7.0	ND	Lee et al., 2010

*ND, not determined; ^∗^, cold active enzyme.*

The activity of the recombinant BpEG was also determined at various temperatures, ranging from 10-60°C at an optimum pH of 6.0. The optimal temperature of BpEG was determined to be 35°C (Figure 4C and Table 2). The result obtained in our study is in accordance with previous reports on endo-1,4-β-glucanase (rPdCel5C) conducted by Liu et al. (Liu et al., 2013). Interestingly, the recombinant BpEG also exhibited about 42% of the catalytic activity at 10°C (Figure 4C). That is to say, BpEG was still functional at low temperatures. Therefore, it suggested that BpEG can be regarded as a cold-adaptive enzyme. In addition, BpEG maintains approximately 80% maximal activity after the treatment at 30°C for 45 min (Figure 4D). That indicated BpEG was relatively stable at low temperatures. The cold adaptability of the recombinant BpEG protein was found to be consistent with the previously reported endo-1,4-β-glucanases (Ueda et al., 2014). These results indicate that BpEG was an enzyme that adapted to a cold environment.

We have listed the previous reports on endo-1,4-β-glucanases from other species in Table 1, and few of them were appraised as cold active endo-1,4-β-glucanases. Namely, the optimal temperatures of the endo-1,4-β-glucanases isolated from the *Penicillium decumbens*, *Citrobacter farmeri* A1 and *E. foetida* were identified between the range of 30 and 50°C, and these enzymes exhibited about 30–40% of their total catalytic activity at 10°C (Ueda et al., 2014; Bai et al., 2016; Liu et al., 2016). In addition, the endo-1,4-β-glucanase (RCE1) isolated from the *Rhizopus oryzae* also exhibited adaptability to low temperatures (Murashima et al., 2002). All these reports suggest that the cold active endo-1,4-β-glucanase had a wide range of distribution. Furthermore, previous reports showed that *Burkholderia* discovered in Antarctic and Arctic lichens, and the lipases of *Burkholderia* were determined as cold-adapted active enzymes (Table 3). Moreover, the optimal temperatures of lipases isolated from *Burkholderia anthina* NT15, *Burkholderia* sp. SYBC LIP-Y and *Burkholderia* sp. JXJ-16 were measured to be 30–35°C (Han and Chen, 2010; Jin et al., 2012; Xie et al., 2017). These results show that *Burkholderia* adapts to cold environments, and their basic metabolism may be sustained at low temperatures. In other words, *Burkholderia* is a very promising producer of cold-active enzymes.

**TABLE 3:** Properties of cold-active enzymes from *Burkholderia*.

**Origin**	**Enzyme name**	**Molecular mass**	**Optimum pH**	**Optimum temperature (°C)**	**pH stability**	**Thermostability**	**References**
*Burkholderia anthina* NT15	Lipase	44.5 kDa	9.5	30	ND	ND	Jin et al., 2012
*Burkholderia* sp. SYBC LIP-Y	Lipase	ND	10	30	3.0–10.5	40–60°C	Yuan et al., 2010
*Burkholderia* sp. JXJ-16	Lipase	ND	8.5–9.0	35	5.0–10.5	30–60°C	Xie et al., 2017
*Burkholderia pyrrocinia* JK-SH007	Endo-1,4-β-glucanase	43 kDa	6.0	35	5.0–8.0	30–50°C	This study

*ND, not determined.*

In order to further characterize BpEG, the enzymatic activity of purified recombinant BpEG was determined to be approximately 11.2 Umg^–1^ against the CMC substrate. The catalytic activity of BpEG was higher than that of *E. coli* (5.2 Umg^–1^ protein) (Park and Yun, 1999). Therefore, the recombinant BpEG represents a more efficient enzyme candidate with various applications, especially in the field of bioconversion. The recombinant BpEG enzymes were not found to be active against the crystalline cellulose substrates containing the 1,4-β-glycosidic bonds. BpEG’s inability to digest the crystalline cellulose was found to be similar to that of endo-1,4-β-glucanase (BcsC) of glycoside hydrolase (GH) family 8 from *E. coli*, *Bacillus circulans* strains and metagenomics study of Ladakh soil (Park and Yun, 1999; Hakamada et al., 2002; Bhat et al., 2013). However, crystalline cellulose is the major substrate for exoglucanases as reported by Han and Chen (2010) and Mahmood et al. (2013). Usually, the enzymatic hydrolysis of insoluble and crystalline cellulose substrates mainly relies on the cellulose-binding domains. Exoglucanase from *S. cerevisiae* has a cellulose-binding domain (Moses et al., 2005), but neither BpEG nor BcsC have designated cellulose-binding domains. Yet these studies demonstrated that BpEG also belongs to the GH 8 family and showed the special endo-1,4-β-glucanase activity against the soluble cellulose substrates.

Thus, we wanted to study the mode of action of the purified recombinant BpEG. We used different substrates including CMC and cello-oligosaccharides of various lengths (cellobiose, cellotriose, cellotetraose, and cellopentaose involved in 1,4-β-glycosidic bonds) to study the hydrolysis rates of BpEG, and the products obtained from the enzyme hydrolysis were analyzed using HPAEC-PAD (Figure 5 and Table 1). Results obtained from the enzymatic CMC hydrolysis experiments of BpEG mostly resulted in glucose (Figure 5B). In addition, the major hydrolysis products from cellopentaose were cellotriose and cellobiose (Figure 5F). A small amount of cellotetraose and glucose production was also observed. The result suggested that BpEG randomly cut cellopentaose, as described by Xu et al. (2000). We also used cellotetraose as a substrate to study the mode of action of the purified recombinant BpEG. The products obtained from the hydrolysis mainly included cellotriose and glucose (Figure 5E). However, our results showed that cellotriose and cellobiose were not degraded (Figures 5C,D). Generally, cellotriose is the substrate for β-glucanase. Results obtained from all our enzyme hydrolysis experiments showed almost similar hydrolysis patterns, and the products obtained from the hydrolysis experiments of endo-1,4-β-glucanases of *E. foetida* (Ueda et al., 2014) were also similar. Therefore, BpEG can be considered as an endo-type 1,4-β-glucanase. These results also suggested that BpEG can degrade cello-oligosaccharides into oligosaccharides with lower polymerization degree.

In order to explore the potential application of BpEG, we used two ways to study. One way is that BpEG degrades the biomasses alone, the release of reducing sugar was limited. The yields of reducing sugars released from wheat bran, oat grain, and ginkgo leaves were 0.042 mg/mg, 0.046 mg/mg, and 0.047 mg/mg in 30 min, respectively. However, the other way is that BpEG and commercial cellulases jointly degrade the biomasses, the released reducing sugar by BpEG from different biomass was increased. The biomass of oat grain exhibited the highest yield of released reducing sugar (100%, 0.22 mg/mg), followed by the ginkgo leaves (45%, 0.10 mg/mg) and wheat bran (32%, 0.071 mg/mg) in 30 min. The yields of reducing sugar from oat grain, ginkgo leaves and wheat bran increased by 5.2, 2.1 and 1.5 folds, respectively. This result is also consistent with that enzymatic hydrolysis of cellulose to glucose is a complicated process, which requires a synergistic action of cellulolytic enzymes including exo-β-1,4-glucanases (EC3.2.1.91), endo-β-1,4-glucanases (EC3.2.1.4), and β-glucosidases (EC 3.2.1.21) (Sharma et al., 2016). Those results suggested that BpEG was a promising candidate in the fields of biofuel, biorefining, food and pharmaceutical industries.

## Conclusion

In this study, we identified and reported on the highly active endo-1,4-β-glucanase (BpEG) isolated from the *B. pyrrocinia* JK-SH007 strain. We implemented an integrated approach by performing a sequence-based bioinformatic analysis and enzymatic hydrolysis and characterization experiments. We have successfully cloned the GC-rich (74.71% of GC) *BpEG* gene. The analysis of the predicted amino acid sequence indicated that BpEG belongs to GH family 8. The *BpEG* gene fragment without a signal peptide coding sequence was successfully expressed in *E. coli* BL21 (DE3). The above-described recombinant BpEG enzyme we obtained was successfully characterized using SDS-PAGE and Western blotting techniques. The stability of BpEG was investigated under various temperatures, pH conditions, and various cellulosic substrates. The optimum pH and temperature of the BpEG were found to be 6.0 and 35°C, respectively. Interestingly, the BpEG retained 42% of its catalytic activity at 10°C, suggesting the BpEG was regarded as a cold-adaptive enzyme. The recombinant BpEG enzyme can play a crucial role in various industrial processes, as it can exhibit significant catalytic activity even at lower temperatures. Thus, the identification of cold-adaptive cellulases, like BpEG, constitutes an important step in the development of a low-temperature dependent industrial process that can achieve synchronous saccharification and fermentation (SSF) process. Additionally, BpEG can degrade various cello-oligosaccharides into oligosaccharides with lower polymerization degrees. That suggested BpEG is a promising candidate to product oligosaccharides for the food industry and pharmaceutical industry. The cold-adaptive recombinant BpEG enzyme can significantly influence various temperature-dependent industrial applications by simultaneously enhancing the cellulose hydrolysis rates.

## Data Availability Statement

The datasets generated for this study can be found in the GenBank database/MH733823.

## Author Contributions

FC, WQ, and JY designed the experiments. FC, XW, and JR performed the experiments. FC and XW analyzed the data. FC, AS, WQ, D-WL, and JY wrote the manuscript.

## Conflict of Interest

The authors declare that the research was conducted in the absence of any commercial or financial relationships that could be construed as a potential conflict of interest.

## Footnotes

1 http://www.cbs.dtu.dk/services/SignalP-4.0/

## Abbreviations

Bcc: *Burkholderia cepacia* complex
BpEG: endo-1,4- β-glucanase from *Burkholderia pyrrocinia* JK-SH007
CBB: coomassie brilliant blue
CMC: carboxymethyl cellulose
dNTP: deoxynucleotide triphosphate
HPAEC-PAD: high performance anion exchange chromatography-pulsed amperometric detector
IPTG: isopropylthio- β-D -galactopyranoside
PASC: phosphoric acid swollen cellulose
SDS-PAGE: sodium dodecyl sulfate-polyacrylamide gel electrophoresis
SSF: synchronous saccharification and fermentation
WB: Western blot.
